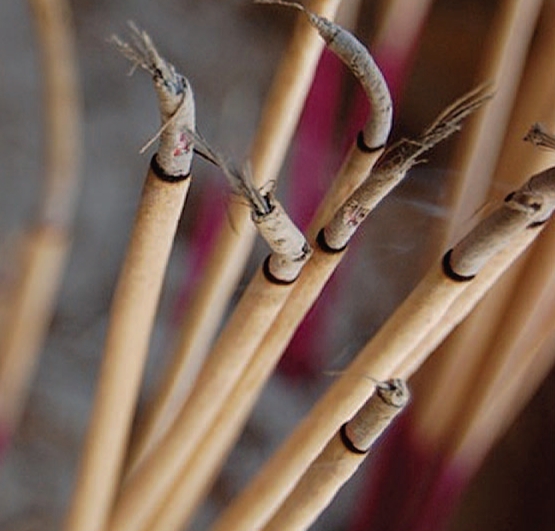# The Beat

**Published:** 2008-09

**Authors:** Erin E. Dooley

## Declaration on Port Pollution

A 2008 UN Intergovernmental Panel on Climate Change report estimates the shipping industry produces nearly 4.5% of the CO_2_ emitted worldwide and that these emissions will increase by 30% by 2020. Shipping emissions are not governed by the Kyoto Protocol, so in July 2008 a group of 55 port officials from 35 countries gathered at the World Ports Climate Conference where they signed the World Ports Climate Declaration, in which parties agree to cut their CO_2_ outputs, possibly by using cleaner-burning fuel, reducing travel speeds, and developing better ship coatings to reduce drag. At the meeting, representatives of the International Maritime Organization announced progress in developing new emissions targets for the shipping industry, to be implemented by 2010.

**Figure:**
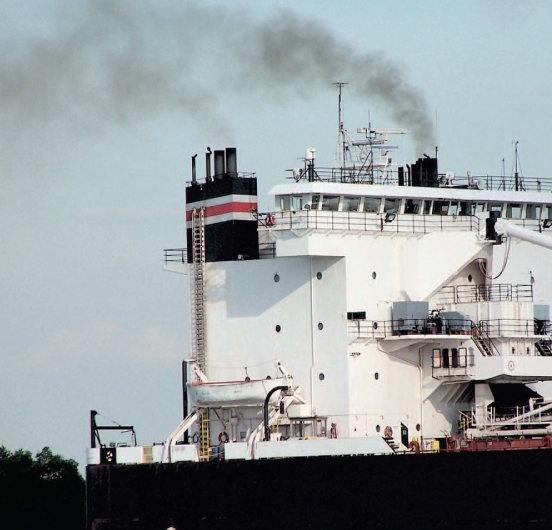


## Keeping Apace with e-Waste

The import of electronic junk into developing countries for recycling or disposal threatens both environmental and human health. Although recycling computers yields valuable metals, improper handling also releases lead, mercury, and other potential toxicants into the environment. At the June 2008 meeting of the Basel Convention on the Control of Transboundary Movements of Hazardous Waste and Their Disposal, parties launched a significant initiative to abate that threat: the Partnership for Action on Computing Equipment, or PACE. The partnership will establish international guidelines for environmentally sound methods of repairing and recycling computer goods, certify facilities that use such methods, and train workers in responsible practices. Parties to the Basel Convention must ensure that hazardous waste is managed in an environmentally sound manner.

**Figure:**
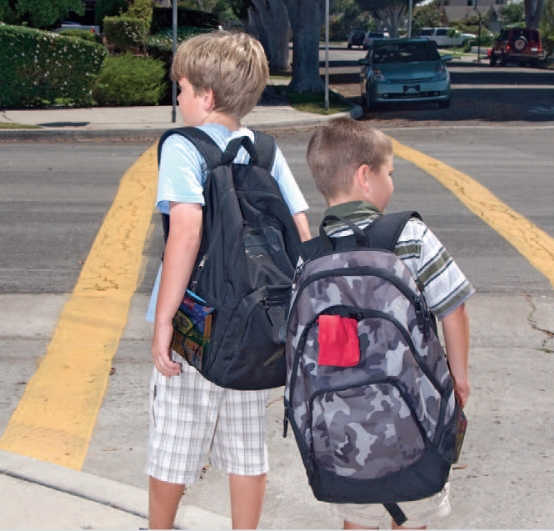


## Walk to School Month 2008

Each October community groups worldwide sponsor events that promote walking to school as a way of improving children’s health, reducing traffic emissions, and enriching community life. In 2007 millions of walkers in 42 countries participated in such events; in the United States, almost 3,000 schools from all 50 states took part. Interested parties can read more about the International Walk to School program at http://www.iwalktoschool.org/, which includes tips for sponsoring events as well as educational materials for teachers, parents, and children.

**Figure:**
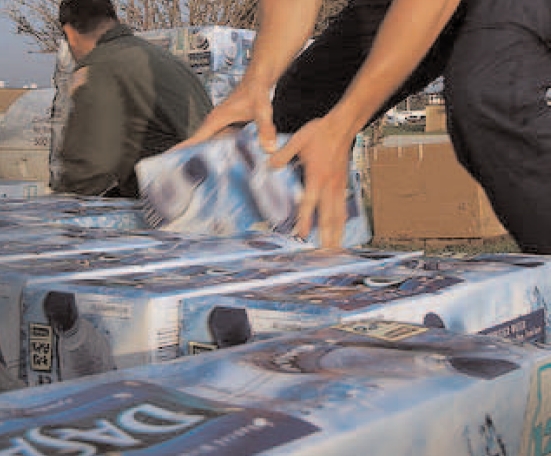
Coast Guard workers distribute bottled water after Hurricane Katrina, 1 September 2005

## Potable Water a Priority in Hurricane Preparedness

In July 2008 the Harvard School of Public Health released survey results on hurricane preparedness of more than 5,000 participants from eight coastal states, plus a special sample of New Orleans residents. Three years after Hurricane Katrina, people affected by the storm named the need for fresh drinking water as a top priority in a storm’s aftermath, and 37% of participants reported keeping water purification supplies on hand. Some 34% of respondents affected by Katrina felt prepared if a major hurricane were to strike their communities within the next 6 months. The Atlantic Ocean hurricane season runs each year from June 1 to November 30.

## Skin Cancer Souvenir?

A population-based study of young, white British women published online 10 July 2008 ahead of print in the *Journal of Investigative Dermatology* suggests that vacationing—but not necessarily living—in hotter or higher-altitude locations than one’s home is associated with a greater whole-body number of nevi (benign moles) in women aged 18–29 who normally live in temperate climates. The association was particularly strong for nevi on the trunk and lower limbs, which typically are only intermittently exposed to the sun. The researchers believe this finding supports the hypothesis that intermittent sun exposure is a primary environmental risk factor for developing nevi, and thus for melanoma. Having a large number of nevi is the strongest known risk factor for melanoma in whites.

## The Health Impact of Incense

In Asian countries where Buddhism and Taoism are the major religions, incense is burned daily in homes and temples. A review published 25 April 2008 in *Clinical and Molecular Allergy* and a study published 9 May 2008 in *Chemico-Biological Interactions* focus on the potential respiratory and carcinogenic effects of incense smoke, which can contain benzene, toluene, xylene, 1,3-butadiene, polyaromatic hydrocarbons, and particulate matter. The first study found that exposure to incense smoke can cause airway dysfunction, elevated cord blood IgE levels, allergic contact dermatitis, and neoplasms, and advises people to reduce exposure when they worship and to ventilate homes during the burning of incense. The second found that temple workers in Thailand had significantly more DNA damage and reduced DNA repair capacity, and warns that exposure to incense smoke may increase the risk of cancer.

**Figure:**